# Human Enamel Fluorination Enhancement by Photodynamic Laser Treatment

**DOI:** 10.3390/polym14142969

**Published:** 2022-07-21

**Authors:** Corina Elena Tisler, Marioara Moldovan, Ioan Petean, Smaranda Dana Buduru, Doina Prodan, Codruta Sarosi, Daniel-Corneliu Leucuţa, Radu Chifor, Mîndra Eugenia Badea, Razvan Ene

**Affiliations:** 1 Department of Prosthetic Dentistry and Dental Materials, “Iuliu Hatieganu” University of Medicine and Pharmacy, 32 Clinicilor Street, 400006 Cluj-Napoca, Romania; tisler.corina@umfcluj.ro (C.E.T.); dana.buduru@umfcluj.ro (S.D.B.); 2 Department of Polymer Composites, Institute of Chemistry “Raluca Ripan”, University Babes-Bolyai, 30 Fantanele Street, 400294 Cluj-Napoca, Romania; marioara.moldovan@ubbcluj.ro (M.M.); doina.prodan@ubbcluj.ro (D.P.); codruta.sarosi@gmail.com (C.S.); 3 Faculty of Chemistry and Chemical Engineering, University Babes-Bolyai, 11 Arany János Street, 400028 Cluj-Napoca, Romania; 4 Department of Medical Informatics and Biostatistics, “Iuliu Hatieganu” University of Medicine and Pharmacy, 6 Pasteur Street, 400012 Cluj-Napoca, Romania; dleucuta@umfcluj.ro; 5 Department of Preventive Dental Medicine, “Iuliu Hatieganu” University of Medicine and Pharmacy, Avram Iancu 31, 400083 Cluj-Napoca, Romania; raduchifor@yahoo.com (R.C.); mindrabadea@gmail.com (M.E.B.); 6 14 Department, Orthopedics, Anesthesia and Intensive Care, University of Medicine and Pharmacy Carol Davila, 37 Dionisie Lupu Street, 020021 Bucharest, Romania; razvan77ene@yahoo.com; 7 Orthopaedics and Traumatology Department, Bucharest Emergency University Hospital, 169 Splaiul Independenței Street, 050098 Bucharest, Romania

**Keywords:** enamel fluorination, photodynamic laser treatment, re-mineralization, roughness

## Abstract

Poor oral hygiene leads to serious damages of theteeth’s surface enamel such as micro-abrasions and acid erosion. These alterations combined with bacterial plaque result in cavity appearance. Prophylactic measures include various techniques for enamel surface restoration. Fluorination is one of the most important treatments for this purpose. Therefore, in the present research, we investigated the classical fluorination treatment compared with laser photodynamic fluorination performed on human enamel samples with poor surface quality. Three sample groups were investigated: veneer (F), inlay (I), and crowns (C). The general morphologic aspect was investigated by scanning electron microscopy (SEM), and the specific details such as the fine microstructure and nanostructure were investigated by atomic force microscopy (AFM) of the surface roughness. The samples were also investigated by Fourier transformed infrared attenuated total reflectance (FTIR-ATR) to evidence the fluorination effect on the enamel surface. Results showed that all initial samples had an altered state with micro-abrasions and erosion with mineral loss, which increase the surface roughness. The F group was the most damaged, having a higher roughness, and the I group was less damaged. Classic fluorination treatment partially restored the enamel by local re-mineralization, but did not obtain the parameters of healthy enamel. However, a significant decrease of the roughness was observed (statistical relevance *p* = 0.001 with the Breusch–Pagan Test). This fact was supported by the presence of newly formed fluorides in the FTIR-ATR spectra. The photodynamic laser fluorination restores the enamel in an enhanced manner by a strong re-mineralization, which implies a significant roughness value decrease comparable to healthy enamel. The Breusch–Pagan Test confirmed the relevance with *p* = 0.001. This is due to an extended re-mineralization abundant in fluoride crystals as observed by AFM and FTIR. Statistical *p*-values regarding laser application were in the range of 0.02–0.06, supporting its relevance in the fluorination effect. The final conclusion is that the photodynamic effect is able to favor the newly formed fluoride deposition onto the affected sites of the enamel surface.

## 1. Introduction

The main strategy to fight against dental cavities is fluorination. Fluoride may be administrated in a general or topical manner depending on the purpose. It ensures good anti cavity protection by enamel re-mineralization. Fluoride is also effective for dental hypersensitivity reduction and presents a significant antibacterial effect according to the data in the literature [1].

Fluoride can be found in various available chemical forms, but calcium fluoride formation agents are the most interesting for the current research. Commercial compound Tiefenfluorid (Humanchemie GmbH, Alfeld, Germany) was successfully used for dental cavity prevention and for enamel re-mineralization. It is available both for children and adults and is used for deeper fluorination of the teeth’s hard tissue. There are only a few studies about the clinical efficiency of this compound [2,3,4,5]. Considering this lack of information, we aim to investigate its efficiency in enamel surface restoration.

There are significant studies in the literature about the effects of laser usage in the fluorination protocols before and after fluoride treatment, evidencing improved fluoride absorption and enamel re-mineralization stimulation [6,7,8,9]. The active compounds within Tiefenfluorid are sodium fluoride and dispersed calcium hydroxide, which are involved in the newly formed fluoride crystals [10,11]. Teng etal. reported that newly formed fluoro hydroxyapatite on the etched enamel ensures a proper protective layer [12].

Charles H. Gerould was the first to certify fluoride’s presence on the surface of dental tissues using electron microscopy in 1945. Since then, several scanning electron microscopy (SEM) studies investigated the ability of fluoride to re-mineralize and modify the enamel surface and the capacity of dental lasers to enhance the topical fluorination effects [13,14,15,16]. Data in the literature report that the surface roughness enhancement due to the laser-assisted fluorination was monitored by atomic force microscopy (AFM) [17]. The most important roughness parameters are *Ra*, which represents the arithmetic average of the profile height, and *Rq*, representing the root mean square of the profile height.

The success of enamel fluorination depends on the proper evaluation of the initial state of the teeth’s surface. Therefore, it is very important to understand the enamel structure and its deterioration mechanisms. Human enamel is a hierarchical structure based on hydroxyapatite nano-crystals embedded in a protein matrix, which ensures cohesion [18]. The enamel nanostructural units have a rounded shape with an average diameter of about 40 nm, which leads to a compact and uniform surface with an *Ra* roughness in the range of 4–8 nm and an *Rq* roughness of about 8–16 nm. These nanostructural units are tightly bonded into fine microstructured prisms with a diameter of about 5 μm. The boundaries between the prisms lead to a significantly rough surface compared to the nanostructure level: *Ra* in the range of 13–20 nm and *Rq* in the range of 25–40 nm [18,19]. We consider these values as the standard for healthy enamel to be compared with our experimental results.

The mechanical effect of mastication may cause accentuated abrasions on the enamel surface if it is associated with different hard particles within the food. Such abrasions increase the surface roughness and facilitate the adhesion of food residues [18,20]. Acidic food and drink consumption presents another important erosive risk by weakening the protein bond within the hydroxyapatite (HAP) nanoparticles, leading to the progressive increasing of the nanostructure unit diameter, ending with disintegration and mineral loss. Such an effect forms washout depressions known as acid erosion features [21,22,23].

Both abrasions and acid erosion result in severe demineralization of the enamel surface. The data in the literature show AFM’s ability to show the morphologic–topographic aspects of demineralization and the progress of the re-mineralization process [24,25]. Therefore, enamel roughness is one of the most important parameters to be monitored in the research on de-mineralization and re-mineralization processes [26,27].

Proper characterization of the initial state of the teeth samples in the present research is very important. All samples were cut from mature teeth, which were used for many years, a fact that may affect the enamel quality in a manner strictly dependent on the oral hygiene during their active period. Some adhesive prosthetic restoration may occur on the investigated surfaces, which supposes a stage of acid engraving of the enamel surface. The initial untreated sample of each considered group was investigated for this purpose, to ensure the optimal demonstration of the morphologic and topographic alteration and to compare with the results obtained on the treated samples.

The objective of the present study is the analysis of the low-power laser diode effect on the fluorination process when it is used after the application of the calcium fluoride formation agent on the teeth’s hard tissues. Our goal is the comparison of the results obtained by simple fluorination and laser photodynamic fluorination by several physical–chemical methods such as SEM, AFM, and FTIR-ATR. Therefore, the null hypothesis is that no difference will occur in the results obtained by both treatments.

## 2. Materials and Methods

### 2.1. Fluorination Protocol

This research used 30 extracted human teeth, *n* = 30, which presented ceramic prosthetic restoration bonded with adhesive. Their use was approved by the Ethics Committee of UMF “Iuliu Hațieganu” Cluj Napoca with No. AVZ14/19.11.2021. The teeth were split equally into three representative groups depending on the restoration mark places: in the veneer Group Fn_f_ = 10; in the inlay area Group In_i_ = 10; at the crown level Group Cn_c_ =10. The ceramic restorations were fabricated from IPS e.max PRESS ceramic ingots using the heat-pressing technique and cemented with adhesive dual-cured cement (Variolink Esthetic DC) Schaan, Liechtenstein. The prosthesis was fabricated immediately after teeth preparation. All preparations were performed by the same dentist; meanwhile prosthodontic restorations were performed by the same dental technician. All sample groups were fluorinated with Tiefenfluorid (Humanchemie GmbH, Alfeld, Germany), which is a bi-component system based on two solutions. The details from the producer are presented in Table 1.

Simple fluorination treatment was performed according to the indications of the producer. The first solution was applied on the medial surface of each tooth using a dental applicator and was kept for 60 s to act. After that, the second solution was applied in the same manner and was left for 5 min to react to complete evaporation. The final step was the washing the fluorinated area with bi-distilled water.

The laser photodynamic fluorination treatment was performed according to the following procedure. The fluorination process was performed in the same manner as the previously described treatment. After the samples were washed, they were irradiated for 180 s with the laser diode SiroLaser Blue (Dentsply Sirona, New York, NY, USA) from the Department of Prevention in Dental Medicine from Iuliu Hatieganu Faculty of Medicine and Pharmacy, Cluj–Napoca, Romania. The laser characteristics were: wavelength 660 nm; power 100 mW, continuous wave (CW), contact mode, power density 400 mW/cm^2^, using a periodontal tip with a 320 μm diameter, attached to the hand piece. The laser procedure was based on our previously established protocols [28]. They are in good agreement with the data in the literature [29,30].Our objective was to assess the effects of low-level laser therapy on fluoridation. We chose the bio-modulation mode of the diode laser (specific for gingival tissue) and explored the way it influences the fluoridation of hard dental tissues, when applied after the calcium fluoride forming agent.

### 2.2. Scanning Electron Microscopy Investigation

SEM analysis was performed with the INSPECT S electron microscope of the FEI Company (Hillsboro, OR, USA), at low vacuum, with an acceleration voltage of 30 kV. SEM images were captured initially and after both treatments at a magnification of ×5000.

### 2.3. Atomic Force Microscopy Investigation

The teeth samples were sliced after the SEM investigation with the microtome IsoMet 100 produced by Buehler, Coventry, U.K. in order to fulfill the requirements of the AFM investigation. The slices were cut in a parallel direction with the enamel surface to ensure an optimal positioning on the AFM sample holder. Each sample group contained a slice from: the initial stage, after fluorination, and after laser photodynamic fluorination. The goal of the present investigation was to reveal the morphological–topographical characteristics at the level of the fine microstructure and nanostructure of the samples.

The AFM investigation was performed with a JEOL JSPM 4210 Scanning Probe Microscopy, Tokyo, Japan. All samples were investigated in tapping mode using NSC 15 cantilevers produced by MikroMasch, Sofia, Bulgaria. The cantilevers’ resonant frequency is about 330 kHz with a force constant of 48 N/m. The topographical images were scanned in an area of 5 μm× 5 μm for the fine microstructure and 1 μm× 1 μm for the nanostructure of the enamel. Three different macroscopic zones on each sample were investigated. The resulting images were processed according to the standard method using the WinSPM 2.0 processing soft, JEOL Tokyo, Japan. There were 2D topographical images for the morphology observation and 3D profiles for the topographical observation with measurements of the *Ra* and *Rq* surface roughness.

### 2.4. Statistical Analysis

The quantitative results regarding the *Ra* and *Rq* surface roughness were subjected to statistical analysis. These were evaluated by quantile–quantile plots. The residual normality was verified for all models, and the presence of heteroskedasticity was tested with the Breusch–Pagan Test. A statistical significance threshold of 0.05 was considered for all tests performed in the present research. All statistical analyses were performed with R version 1.1.2 Core Team. R: A Language and Environment for Statistical Computing, Viena, Austria.

### 2.5. FTIR-ATR Investigation

The samples were analyzed with an FTIR spectrometer (Jasco 610, Jasco International Co., Ltd., Tokyo, Japan) in ATR mode, with a scanning range from 4000 to 550 cm^−1^ at a speed of 4 cm^−1^ s ^−1^ and with an average of 128 measurements in the final spectrum.

## 3. Results

### 3.1. Scanning Electron Microscopy

**Figure 1:**
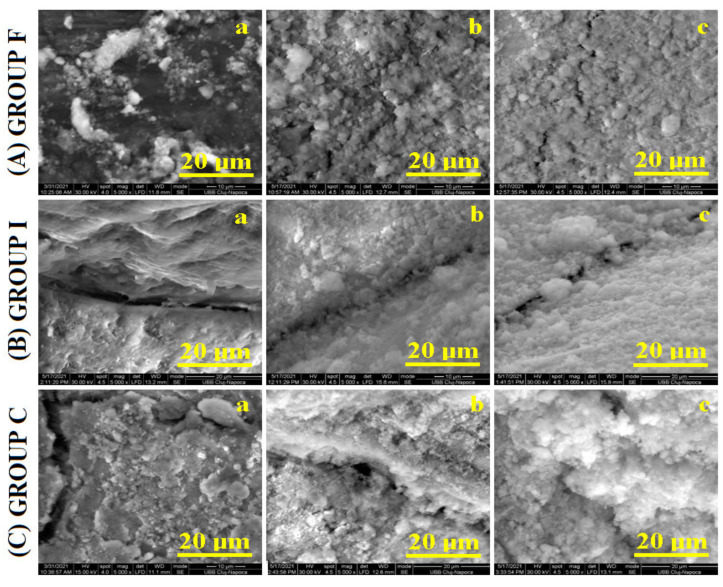
SEM images of the enamel samples: without treatment (**a**), after fluorination (**b**),and after photodynamic laser fluorination (**c**) for the investigated samples.

The general aspect of the samples was investigated by SEM, and the resulting images are presented in Figure 1. The morphology of the initial sample was complex, featuring a combination of abrasion lesions and acid erosion with the occurrence of several cracks.

The veneer sample’s initial morphology is presented in Figure 1A(a); it reveals a highly eroded enamel with many acid erosion depressions and several marks of abrasion. The inlay group reveals a significant gap formed on the border between the enamel and inlay; see Figure 1B(a). Next to this gap, both the enamel and inlay surfaces were not very affected by teeth wear. The crown group revealed a very affected initial surface (Figure 1C(a)) having some significant cracks on the left side of the image and eroded features in the center. It is very difficult to establish which of the initial samples was more affected. The morphological aspects indicate that the crown and veneer groups were more affected by erosion than the inlay area.

SEM images for the samples treated by simple fluorination revealed good morphological improvement by the generation of a new mineralized layer. The general tendency was to fill the deeper areas such as scratches and erosion depressions. A good coverage was observed for the veneer group (Figure 1A(b)), where the newly formed mineral covered the main eroded areas. Figure 1B(b) shows the tendency to fill the gap between the enamel and inlay to form a compact layer. A weaker coverage was obtained for the crown group (Figure 1C(b)), where a significant scratch appears to be uncovered.

The laser-assisted fluorination treatment proved to be more effective, as observed in the SEM images. Figure 1A(c) evidences the enhancement obtained in the veneer group. There appears a very good penetration of the fluoride into the deeper areas such as scratches and covering the eroded area in a uniform manner. The inlay group (Figure 1B(c)) showed a good cover of the gap between the enamel and inlay and an increased tendency of surface smoothing. The crown group presented also satisfactory restoring of the enamel surface by a proper coverage of all initial faults by the newly formed mineral layer; see Figure 1C(c).

SEM investigation proved that the sample morphology resulting after photodynamic laser fluorination was far better than simple fluorination. This means that light irradiation improves the crystallization of the newly formed fluorides. SEM observation indicated that the morphological improvements started on the finest structural details, which were further propagated over the whole sample surface. Therefore, a more detailed microscopic investigation was required.

### 3.2. Atomic Force Microscopy

The AFM investigation was focused on two important microscopic details. The first one is represented by the fine microstructure observed on the surface of an HAP prism unit at a scanning area of 5 µm × 5µm. The second one is represented by the enamel surface nanostructure in the inner area of an HAP prism.

**Figure 2:**
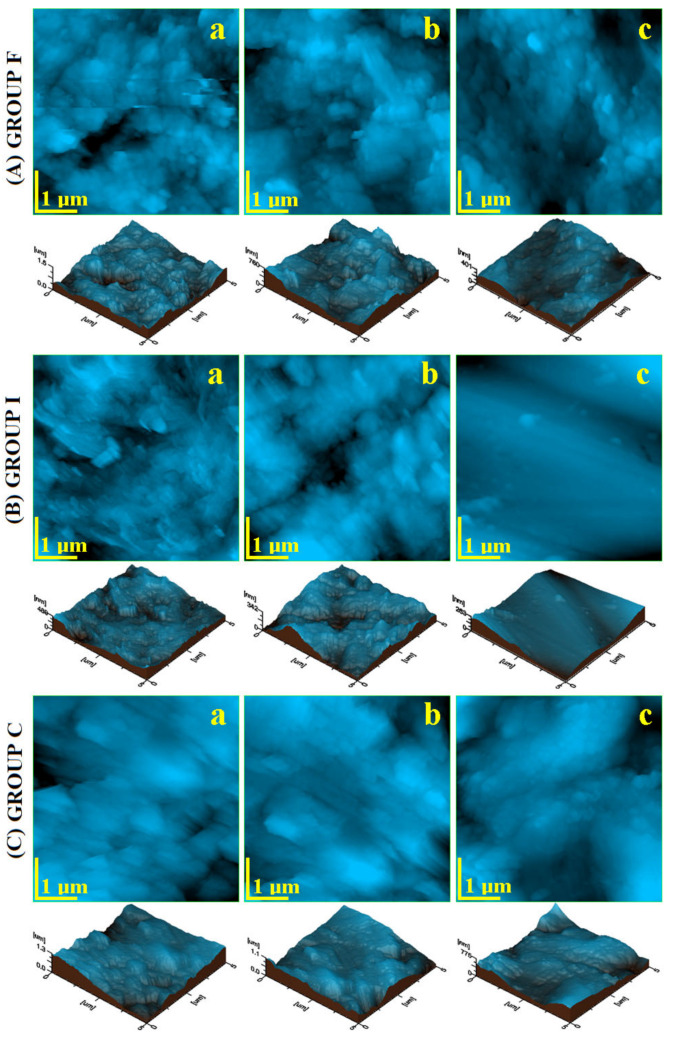
AFM topographic images of the fine microstructure without treatment (**a**), after fluorination (**b**), and after photodynamic laser fluorination (**c**), for the investigated samples. The tridimensional profile is given below each topographic image.

**Figure 3:**
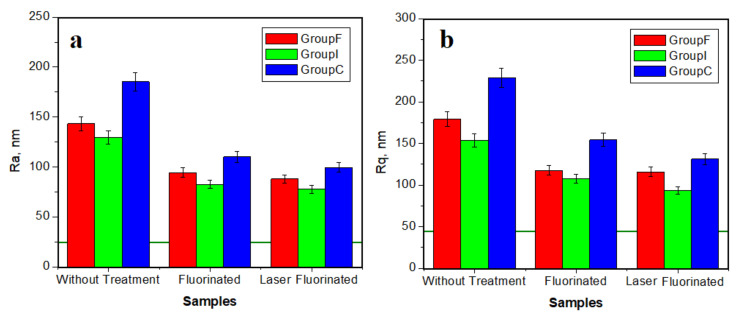
Average roughness variation with the applied treatment for the fine microstructure: (**a**) *Ra* and (**b**) *Rq*. The horizontal green line on the histograms represents the typical value for healthy enamel.

The fine microstructure of the F group (Figure 2A(a)) presented a very irregular topography, having deep marks of mechanical abrasion combined with acid erosion features. This led to a rough surface, as observed in the 3D profile, given below the image, with a local height of 1500 nm. The I group presented a surface that was moderately affected by abrasion and acid erosion. However, the gap between the enamel and inlay contained many submicron debris conglomerates as a secondary result of mineral loss. Therefore, the height observed in the 3D profile was only about 439 nm. The initial surface of the C group evidenced a very irregular fine microstructure with certain abrasion traces (scratches oriented relatively parallel at a 45° angle) (Figure 2C(a)) combined with acid erosion features with submicron clusters of about 150–900 nm in diameter. Definitely, this is a rough surface, as observed in the 3D profile, where the local height roseto1300 nm. The roughness values are presented in Figure 3.

The simple fluorination treatment led to a certain improvement of the fine microstructure for each group. The obtained result depends to a great deal on the initial sample quality. The F group showed a strong attenuation of the abrasion marks due to the newly formed fluoride minerals filling these gaps, but some eroded features were still visible, Figure 2A(b). The relative height decreased to 760 nm, which should result in the roughness decreasing. The inlay group showed a fine microstructure with certain improvements of the fine microstructure; see Figure 2B(b). The erosion features were well covered with the newly generated mineral layer; the debris within the gap was removed, and it was partly filled up with the newly generated fluoride crystals. The resulting local height was about 439 nm.

The crown group after simple fluorination revealed a well improved surface topography; see Figure 2C(b). The newly formed crystals’ proliferation attenuated the abrasion marks and filled the eroded area in order to form a more uniform surface. Besides the evident improvements of the C group, the topography was still far from that typical of healthy enamel.

Photodynamic laser fluorination treatment proved to be more efficient than simple fluorination. The enamel fine microstructure was restored in a more advanced manner. The topography of the veneer group (Figure 2A(c)) revealed that the abrasion marks were completely removed and the structural formations were sharper because of the eroded areas’ coverage with the new mineral addition. The3D profile was smoother, and the local height was situated at only 401 nm. The photodynamic effect of the laser irradiation after the inlay group’s fluorination resulted in advanced restoration of the surface, which was very smooth and compact. The inlay gap was completely filled up with newly formed fluoride crystals, a fact better observed in the 3D profile. The local height was about 263 nm, very low compared with the initial sample. Similar improvements were observed for the crown group; see Figure 2C(c). The abrasion marks are well covered and the eroded areas refreshed by the uniform deposition of the new fluoride nanoparticles. The aspect of the 3D profile featured a local height of about 401 nm and was considerably smoother than the one observed for simple fluorination.

The revealed topographical aspects proved a good improvement of the enamel surface after simple fluorination with Tiefenfluorid and an excellent restoration after the photodynamic laser fluorination. It seems that a synergy occurs between the fluoride formation process and laser irradiation. The success of the effectuated treatments can be quantitatively appreciated by surface roughness monitoring. We followed the most important two roughness parameters, which are *Ra* and *Rq*. *Ra* represents the arithmetic average of the profile height and is described by Equation (1), and *Rq* represents the root mean square of the profile height and is described by Equation (2):

(1) $$Ra=\frac{1}{l_{r}}\int_{0}^{l_{r}}\left|z\left(x\right)\right|dx,$$

and

(2) $$Rq=\sqrt{\frac{1}{l_{r}}\int_{0}^{l_{r}}\left|z{\left(x\right)}^{2}\right|dx}.$$

where: *l* is the profile length and *z* is the height at the *x* point. Both *Ra* and *Rq* are important for various research applications. Therefore, we followed the variation of both roughness parameters during the performed treatments. The resulting values for the fine microstructure are presented in Figure 3.

The green horizontal line in Figure 3 represents the typical value for healthy enamel measured with AFM according to the data in the literature. The resulting roughness for the initial samples was situated over 125 nm for *Ra* and 150 nm for *Rq*, considerably higher than for healthy enamel, a fact in good agreement with the morphologic–topographic observation in Figure 1 and Figure 2. The roughest samples were in the crown group, and the least rough samples belonged to the inlay group.

The roughness significantly decreased after simple fluorination due to the topographic improvements observed by AFM. The resulting values for the veneer and inlay group decreased below 100 nm for *Ra* and 130 nm for *Rq*, smoothing the surface. The values for the crown after simple fluorination were smaller than in the initial stage, but still increased, a fact in good agreement with morphological observation.

A more enhanced roughness decrease was observed after dynamic laser fluorination treatment. The values decreased below 100 nm for *Ra* and 130 nm for *Rq* for all sample groups. This is quantitative proof of the surface smoothing and the success of the applied treatment.

**Figure 4:**
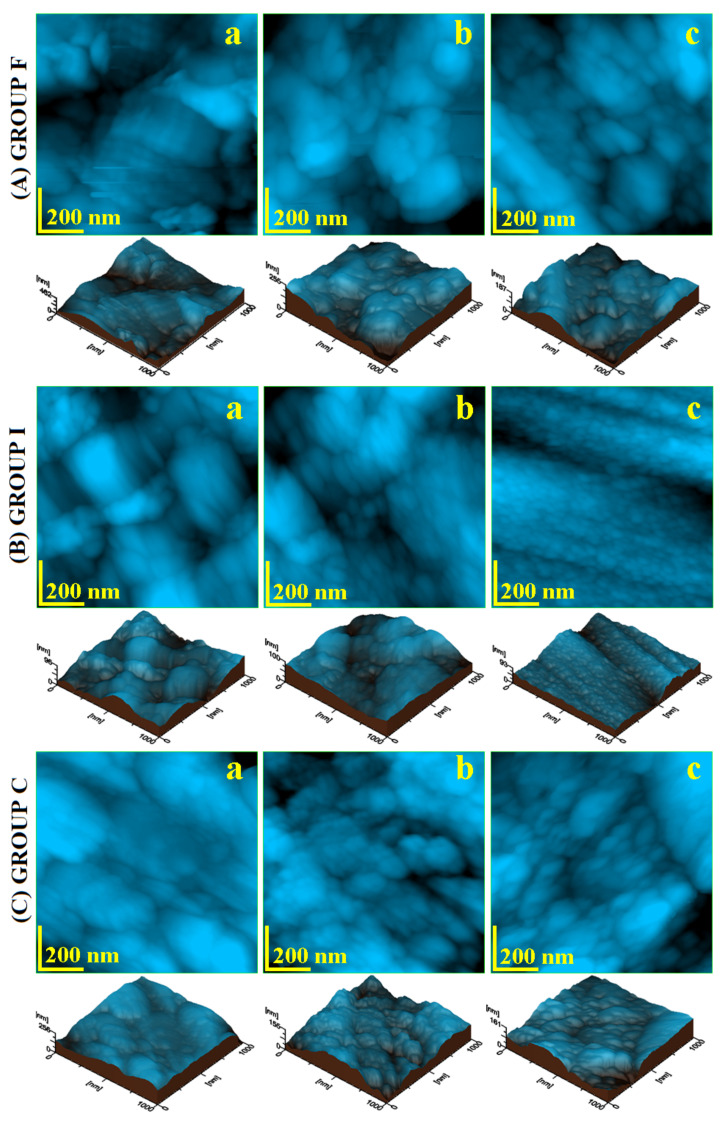
AFM topographic images of the nanostructure without treatment (**a**), after fluorination (**b**), and after photodynamic laser fluorination (**c**), for the investigated samples. The tridimensional profile is given below each topographic image.

The enamel nanostructure was investigated by AFM by surface topography scanning in an area of 1 µm × 1 µm. The resulting images are presented in Figure 4. The initial samples had an altered aspect due to the mechanical abrasion combined with acid erosion. The veneer group showed deep abrasion marks with edge washout by acid erosion; see Figure 4A(a). Their nanostructural units were altered, presenting an average diameter of about 94 nm. The 3D profile evidenced the irregular topography having a local height of 462 nm. The inlay group nanostructure was very affected by acid erosion, with significant mineral loss, and less affected by abrasion; see Figure 4A(b). Therefore, submicron clusters occurred on the surface due to the mineral loss debris having dimensions in the range of 200–600 nm. The nanostructural units can be observed in the areas without erosion debris and presented an average diameter of 59 nm. Besides all the decay features, the local height in the 3D profile was situated at 96 nm. The crown group revealed an altered nanostructure affected by both erosive factors. The abrasion scratches were attenuated by intense acid erosion. The nanostructural units were affected by the weakening of the protein bond, a fact leading to an average diameter of about 68.33 nm.

Simple fluorination treatment resulted in a visible improvement of the nanostructure topography. Figure 4A(b) reveals a well-restored surface by the proliferation of the formed fluoride nanoparticles in the veneer group. Their average diameter was situated around 57.66 nm and covered the abrasion marks and eroded areas in a uniform and compact manner. The inlay group after fluorination featured newly formed fluoride nanoparticles with a diameter of about 48.33 nm, very close to the healthy enamel nanostructural units; see Figure 4B(b). They were well bonded together in a compact structure that covered the eroded enamel. Figure 4C(b) reveals the uniform manner in which the newly formed fluoride nanoparticles covered the eroded surface of the crown sample group. They had an average diameter of 45 nm, very close to the healthy enamel nanostructural units, and presented a refreshed aspect of the 3D profile.

Photodynamic laser fluorination resulted in a more enhanced restoration of the enamel nanostructure. The newly formed fluoride nanoparticles were more numerous than after simple fluorination and ensured a more compact and smoother surface. The veneer group presented newly deposited mineral nanoparticles with a diameter of about 50.33 nm that were very well bonded together; see Figure 4A(c). The synergy between fluorination and laser irradiation is better illustrated by the results observed for the inlay group; see Figure 4B(c). Parallel rows of newly formed mineral nanoparticles appeared, having diameters of about 41 nm that very well bonded together in a compact surface.

The crown group presented a very well improved nanostructure; see Figure 4C(c). The newly formed fluoride nanoparticles had a diameter of 43.66 nm and were very well embedded into the enamel surface, ensuring a proper coverage of the eroded areas.

**Figure 5:**
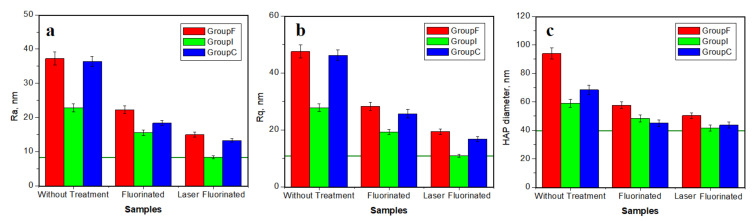
Average roughness variation with the applied treatment at the nanostructure level: (**a**) *Ra*, (**b**) *Rq*, and (**c**) nanostructural unit diameter. The horizontal green line on the histograms represents the typical value for healthy enamel.

All topographic and morphologic aspects from Figure 4 ha a great impact on the surface roughness variation; see Figure 5. The *Ra* variation is presented in Figure 5a and the *Rq* variation in Figure 5b. The poor quality of the initial enamel surface caused by the abrasion and acid erosion led to increased values of the roughness. The rougher samples at the nanostructural level were: the veneer group followed closely by the crown group, the inlay group presenting a moderate roughness. The simple fluorination treatment resulted in a significant reduction of the roughness. The value for the veneer group decreased in a significant manner, comparable with the initial roughness of the inlay samples. Laser fluorination enhances the roughness, decreasing it in a proactive manner. The veneer group’s roughness after the photodynamic treatment was similar to the inlay group’s roughness after simple fluorination. The roughness obtained for the inlay group was similar to the one characteristic of healthy enamel.

The roughness evolution trend with the applied treatments was proportional for all tested groups; the difference was introduced by the roughness of the initial sample. Therefore, a statistical analysis was necessary.

Nanoparticle diameter variation with the applied treatment is presented in Figure 5c. The acid erosion caused bigger diameters in the initial samples. The generation of the newly formed mineral nanoparticles improved the surface quality with newly added nanoparticles with diameters close to the one’s characteristic for healthy enamel. The strongest resemblance to healthy enamel was obtained by the photodynamic laser treatment.

### 3.3. Statistical Analysis Results

The simple and multiple linear regression models were realized for *Ra*, *Rq*, and Dp as dependent variables; the values are given as the logarithm because they do not follow a normal distribution. The models included the lots F, I, and C as independent variables, as well as the sets: (nano vs. micro); fluoride utilization (yes vs. no); LASER utilization (yes vs. no). The presence of multi-collinearity was evaluated for the multivariate models by the inflation factor of the variance. The regression results are presented through the regression coefficient, the trust interval of 95% (calculated with the sandwich estimator method), and the result of the statistical significance of the coefficient. Each independent variable’s effect on the dependent variable is presented:

Multiple regression: natural logarithm of *Ra* (nm) as a function of Lot+SET+Fluor+LASER. For testing the statistical significance of the model results, *p* ≤ 0.001 (statistical F = 113.35 with 5, 48 g.d.l.). The model has a residual standard error = 0.13, a determination coefficient = 0.92, and an adjusted determination coefficient = 0.91. The relevant values aregivenin Table 2 and Table 3. The regression model with robust standard errors (using sandwich package HC1)–enter:

Multiple regression: natural logarithm of *Rq* (nm) as a function of Lot+SET+Fluor+LASER. For testing the statistical significance of the model results, *p* ≤ 0.001 (statistical F = 91.45 with 5, 48 g.d.l.). The model has a residual standard error = 0.14, a determination coefficient = 0.90, and an adjusted determination coefficient = 0.90. The relevant values are given in Table 2 and Table 3. The regression model with robust standard errors (using sandwich package HC1)–enter:

Multiple regression: natural logarithm of Dp (nm) as a function of Lot+SET+Fluor+LASER. For testing the statistical significance of the model results, *p* < 0.001 (statistical F = 7.88 with 4.22 g.d.l.). The model has a residual standard error = 0.09, a determination coefficient = 0.59, and an adjusted determination coefficient = 0.51. The relevant values are given in Table 4 and Table 5. The regression model with robust standard errors (using sandwich package HC1)–enter:

The overall aspects revealed by the statistical analysis show that the nanostructural roughness is independent of the microstructural roughness, but the latter is dependent on the former. The veneer and crown lots featured almost the same roughness range, while the inlay lot presented different values. This resulted from the AFM and SEM investigation. Another important aspect revealed by the statistical analysis is the importance of the “fluoride” parameter on the roughness variation: if it is yes, the roughness decreases; if it is no, the roughness remains high. The models revealed that the “LASER” parameter is also very important.

### 3.4. FTIR-ATR

**Figure 6:**
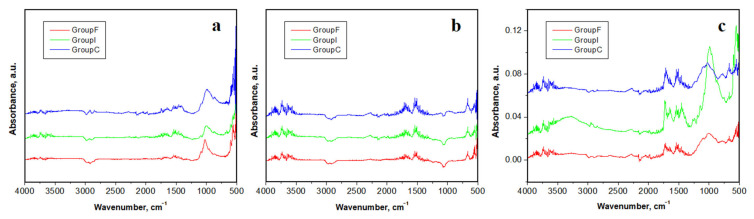
FTIR-ATR spectra for the enamel samples: (**a**) initial untreated, (**b**) after fluorination, and (**c**) after photodynamic laser fluorination.

The FTIR-ATR analysis is the most recommended for new fluoride formation on the enamel surface because of its ability to investigate the chemical bonding vibrational bands. The resulting spectra are presented in Figure 6, and each vibration band was analyzed.

All peaks identified in the FTIR-ATR spectra in Figure 6 are characteristic of the biological carbogaseous hydroxyapatite, found in enamel. The most intense peaks correspond to the symmetrical stretching vibration (ν_1_ mode) of phosphate (PO_4_^3−^), then for carbonate mode (CO_3_^2−^), which overlaps the phosphate band ν_3_.

All the peaks belonging to the mineral content are evidenced in the spectra within Figure 6 in the range of 550–667 cm^−1^ for the band (ν4 phosphate vibration); 1000–1130 cm^−1^ (ν3 phosphate vibration). There were also the peaks identified for the organic compounds within the samples: 852 cm^−1^ and 1062 cm^−1^ (carbonate); 1440 cm^−1^ (amide II and carbonate—modes CH_2_ and NH), 1670 cm^−1^ (amide I), and 2944 cm^−1^ (C–H stretching mode).

The weaker bands identified at 1003, 667, and 566 cm^−1^ were attributed to the phosphate ions belonging to the hydroxyapatite according to Carvalho et al. [31].

The bands observed at 1676, 1458, and 1253 cm^−1^ were attributed to collagen according to Martinez et al. and Khalid et al. [32,33], and the bands 1076, 965, 588, and 432 cm^−1^ were attributed to the phosphate ions within hydroxyapatite [31].

The phosphate absorption FT-IR bands’ analysis in the range of 1030–1090 cm^−1^ revealed the different crystallinity grade between the analyzed enamel samples. In fact, the absorption bands reported in Figure 6b demonstrate a low resolution level of the phosphate peak for simple fluorinated enamel. On the other side, the obtained FT-IR bands for the enamel treated by fluorination showed the presence of a peak at 3573 cm^−1^, which belongs to the hydroxyl groups’ substitution with the fluoride ions in the hydroxyapatite structure, in good agreement with the literature [34]; this peak is visible in Figure 6b.Such a complex interaction of fluoride ions has been observed for several dental materials, such as fluorinated methacrylate [35], fluorinated polyimide [36], and low-fluorinated oligoamides [37]. The photodynamic laser treatment effect on the enamel samples (Figure 6c) did not cause the appearance of new bands or the disappearance of some bands, in good agreement with the data in literature evaluating other laser wavelengths on dental tissues [38,39,40,41].

## 4. Discussion

Dental cavities are the most common oral disease. The fluorinating agents used to prevent cavity occurrence comprise a successful solution with proven efficiency, being a widely applied method. These agents placed on dental tissues generate newly formed calcium fluoride, which is able to release ions under an acid pH [13].

Tiefenfluorid is such a kind of CaF_2_ formation agent. It presents a complex action mechanism that ensures a deep fluorination because of the smaller size of the calcium fluoride precipitate crystals, which is about of 50 Å. The first solution enters into the widened interprismatic spaces, and the second solution, which contains Ca(OH)_2_, interacts with the initial, smaller CaF_2_ particles to finally form the desired calcium and magnesium fluoride crystals [2].

The action is time dependent according to the data in the literature to obtain the optimal structure, which is about 6 min [2,13]. The application protocol and the deep penetration of the product into the abrasion features and acid erosion depressions were proven by the AFM and SEM investigations.

Enamel surface re-mineralization was observed after the simple fluorination as a new layer of nanoparticles generated by the calcium and magnesium fluoride crystals produced by the Tiefenfluorid reactions. They were uniformly distributed on the surface, filing the eroded area, supported by the significant decrease of the surface roughness. Their diameter depends on the Tiefenfluorid solution’s interactions with each sample group: 57.66 nm for veneer; 48.33 nm for inlay; 45 nm for crown.

Data in the literature show that a blue laser is able to activate polymerization in dental composites [42], and it may involve the light curing of the enamel surface treatment [43]. Therefore, light irradiation is able to give a boost the chemical reactions within the Tiefenfluorid reagents during fluoride formation. The results obtained in the present research prove that photodynamic laser fluorination enhances the reactions on the sample surface, as well as in the deeper eroded areas for better nanoparticle proliferation. The newly formed layer has a greater density of particles on the surface, resulting in a more uniform coverage. This fact is supported by the strong decrease of the surface roughness, the nanostructural values being very close to healthy enamel. Their diameter also depends on the interaction with each group: 50.33 nm for veneer, 41 nm for inlay, and 43.66 nm for crown. These values are consistent with the data in the literature [44,45]. Tiefenfluorid’s restoration of the enamel surface is comparable with some of our previous results [46,47].

The null hypothesis states that the photodynamic treatment had no effect on the enamel fluorination effect, but the obtained results reject this. The direct consequence is that the effect fluorination treatment is enhanced by the laser irradiation of the samples after the classic protocol was applied. The light curing during the fluoride crystal formation proved to be effective with respect to their growth. The limitation of the present study is that only the laser was used after fluorination with Tiefenfluorid according to the producer’s instructions. Further research might use some chemical additives to be combined with the laser procedure in order to facilitate a more intense fluoro-hydroxyapatite generation.

The Tiefenfluorid chemical interaction with the enamel composition was investigated by FTIR-ATR. The spectra of the initial sample mainly revealed vibration bands for phosphates due to the hydroxyapatite from the enamel structure and some organic bonds related to the collagen bonds of the HAP nano-crystals. ATR spectra after fluorination revealed significant extra bands related to calcium fluoride formation; there were also some low-intensity bands, which may suggest the occurrence of fluoro-hydroxyapatite as a direct reaction of NaF from the first solution of Tiefenfluorid with Ca_5_(PO_4_)_3_(OH)from the enamel. This chemical mechanism is supported by the data in the literature [12,48,49]. The reaction is presented in Equation (3). The fluoride-related vibration bands, as well as the ones corresponding to the fluoro-hydroxyapatite were more intense for the photodynamic-laser-fluorinated samples. This is in good agreement with the microstructural and nanostructural investigation performed with AFM and SEM.

Ca_5_(PO_4_)_3_(OH) + NaF→Ca_5_(PO_4_)_3_F + NaOH (3)

The incipient formation of the fluoro-hydroxyapatite upon contact of Tiefenfluorid enhances the cohesion of the newly formed fluorides with the hydroxyapatite from the enamel. This is supported by the profound restoration of the nanostructure mainly after laser-assisted fluorination.

The fluoride ion release from the newly formed mineral nanoparticles under an acid pH provides a permanent protective shield. It may enhance the cohesion of this protective layer to the basal layer of the natural hydroxyapatite by the promotion of an inter-layer of fluoro-hydroxyapatite considering the chemical Equation (4):

Ca_5_(PO_4_)_3_OH + F→Ca_5_(PO_4_)_3_F + OH (4)

It is too early to evidence this chemical mechanism using the current samples because they were not exposed to the acid environment, but this may be an interesting direction for future research. However, there are some studies in the literature that describe this reaction as promising [50,51].

## 5. Conclusions

The present research evidenced that long-time wear of teeth combined with poor oral hygiene results in significant alteration of the enamel surface. The abrasion marks combined with acid erosion depressions and mineral loss lead to an increased roughness of the enamel surface. The use of Tiefenfluorid in the simple fluorination process results in a good surface restoration proven by the significant roughness decrease. This is due to the deeper penetration of the Tiefenfluorid reagents and its ability to form new mineral nanoparticles to regenerate the samples’ nanostructures and microstructures. The photodynamic laser applied to Tiefenfluorid fluorination enhances the newly mineral nanoparticles’ proliferation, supported by the best roughness values obtained for the nanostructure, which were similar to healthy enamel. The results showed that the laser photodynamic treatment may be applied to patients after the classic Tiefenfluorid fluorination is performed. The benefit will be a better restoration of the affected areas.

## Figures and Tables

**Table 1:** Tiefenfluorid composition from the producer data sheet.

Compounds of the First Solution	Compounds of the Second Solution
Copper hexafluorosilicate (CuF_6_Si)	Dispersed calcium hydroxide (Ca(OH)_2_)
Magnesium hexafluorosilicate (F_18_Mg_16_Na_10_O_66_Si_27_)	
Sodium fluoride (NaF)	Methylcellulose
Distilled water	Distilled water

**Table 2:** Roughness multivariate model coefficients.

	*Ra*			*Rq*		
	B	(95% CI)	*p*	B	(95% CI)	*p*
(Intercept)	2.27	2.19	<0.001	2.38	2.30	<0.001
Lot (F vs. C)	−0.02	(−0.1–0.05)	0.562	−0.05	(−0.14–0.04)	0.262
Lot (I vs. C)	−0.15	(−0.23–−0.08)	<0.001	−0.19	(−0.29–−0.1)	<0.001
Set (nano vs. micro)	−0.76	(−0.83–−0.69)	<0.001	−0.74	(−0.82–−0.67)	<0.001
Fluoride (yes vs. no)	−0.23	(−0.32–−0.14)	<0.001	−0.2	(−0.3–−0.1)	<0.001
LASER (yes vs. no)	−0.1	(−0.19–−0.01)	0.036	−0.12	(−0.22–−0.02)	0.024

**Table 3:** Robust univariate submodels’ generation.

	*Ra*				*Rq*			
	B	(95% CI)	*p*	R2	B	(95% CI)	*p*	R2
Lot (F vs. C)	−0.02	(−0.3–0.25)	0.87	0.025	−0.05	(−0.32–0.22)	0.705	0.038
Lot (I vs. C)	−0.15	(−0.45–0.14)	0.314	0.025	−0.19	(−0.49–0.1)	0.194	0.038
Set (nano vs. micro)	−0.76	(−0.86–−0.65)	<0.001	0.793	−0.74	(−0.85–−0.63)	<0.001	0.771
Fluoride (yes vs. no)	−0.28	(−0.5–−0.06)	0.016	0.095	−0.26	(−0.48–−0.04)	0.024	0.083
LASER (yes vs. no)	−0.21	(−0.46–0.04)	0.102	0.055	−0.22	(−0.47–0.03)	0.091	0.06

**Table 4:** Multivariate model coefficients: Dp.

	B	(95% CI)	*p*
(Intercept)	1.82	1.72	<0.001
Lot (F vs. C)	0.1	(0.02–0.19)	0.03
Lot (I vs. C)	−0.01	(−0.1–0.07)	0.735
Fluoride (yes vs. no)	−0.16	(−0.25–−0.06)	0.004
LASER (yes vs. no)	−0.04	(−0.11–0.03)	0.259

**Table 5:** Robust univariate submodels’ generation: Dp.

	B	(95% CI)	*p*	R2
Lot (F vs. C)	0.1	(−0.02–0.23)	0.124	0.165
Lot (I vs. C)	−0.01	(−0.12–0.09)	0.781	0.165
Fluoride (yes vs. no)	−0.18	(−0.28–−0.08)	0.002	0.407
LASER (yes vs. no)	−0.12	(−0.2–−0.04)	0.006	0.188

## Data Availability

The data presented in this study are available on request from the corresponding authors.
